# Factors associated with kindergarten teachers’ willingness to continue working

**DOI:** 10.1097/MD.0000000000027102

**Published:** 2021-09-03

**Authors:** Moemi Matsuo, Goro Tanaka, Akiko Tokunaga, Toshio Higashi, Sumihisa Honda, Susumu Shirabe, Yuri Yoshida, Akira Imamura, Izumi Ishikawa, Ryoichiro Iwanaga

**Affiliations:** a Faculty of Rehabilitation Sciences, Nishi Kyushu University, 4490-9 Ozaki, Kanzaki, Saga, Japan; b Unit of Medical Science, Nagasaki University Graduate School of Biomedical Sciences, 1-7-1 Sakamoto, Nagasaki, Japan; c Center for Child Mental Health Care and Education, Nagasaki University, 1-7-1 Sakamoto, Nagasaki, Japan; d Organization of Rural Medicine and Resident Education, Nagasaki University Hospital, 1-7-1 Sakamoto, Nagasaki, Japan; e Faculty of Education, Nagasaki University, 1-14 Bunkyo, Nagasaki, Japan; f Unit of Neuropsychiatry, Nagasaki University Graduate School of Biomedical Sciences, 1-7-1 Nagasaki, Japan.

**Keywords:** job satisfaction, mental health, preschool teacher, turnover, work engagement

## Abstract

The turnover rate among kindergarten teachers in advanced countries is extremely high. As such, there is an urgent need to determine the reasons for this turnover and to identify ways to prevent it. The current study investigates the individual and environmental factors that impact kindergarten teachers’ willingness to continue working.

A total of 600 kindergarten teachers in Japan participated in this study. Participants responded to questionnaires regarding their willingness to continue working, mental health, work engagement, and the availability of social support. Multiple logistic regression analysis was used to analyze participants’ data, with willingness to continue working for 5 or more years as the dependent variable. Additionally, Spearman rank correlation was used to examine the correlations between factors associated with willingness to continue working.

Factors such as older age, living with a spouse, caring for younger children (up to 2 years old) at work, good mental health, and higher work engagement were significantly associated with teachers’ higher willingness to continue working. Factors such as marriage, health and family problems, overtime work, issues with workplace childcare, and education policy, working time/day problems, human relations, and difficulties taking care of children were correlated with teachers’ lack of willingness to continue working.

The findings of this cross-sectional study suggest that welfare benefits and individual support systems could be key elements to encourage kindergarten teachers to continue working and could lead to their improved job satisfaction and mental health. Balanced work conditions and workers’ high agreement with their workplace's overall childcare or educational policies may lead to lower turnover. Some programs – such as relationship counselling – could have a positive impact on teachers’ mental health and job satisfaction.

## 1 Introduction

The turnover rates of kindergarten teachers in advanced countries such as Japan and Sweden are extremely high; hence, there is an urgent need to determine the reasons for this turnover and identify ways to prevent it.^[1,2]^ Moreover, Japan has been facing a shortage of kindergarten teachers,^[3]^ resulting in children receiving insufficient care in kindergartens and parents not letting their children attend, which affects parents’ ability to work. Some have shown a reduced inclination to have children because of this social issue, contributing to the fertility decline in Japan and making it imperative to reduce turnover rates according to Ministry of Health, Labour and Welfare, Japan.

Researchers have identified various factors, including individual and environmental factors that impact kindergarten teachers’ intention to continue working. In a meta-analysis, demographic and job characteristics, and job satisfaction were significantly associated with the turnover intention of workers in 1 study.^[4]^ Another study conducted by researchers at the authors’ university investigated factors impacting the turnover rate among kindergarten teachers, showing that gender, age, mental health, social support, and work engagement were associated with teachers’ willingness to continue working.^[3]^ In terms of individual factors, higher job satisfaction has been shown to be positively related to teachers’ mental health, and higher job satisfaction and work engagement have been shown to lower turnover rates.^[2,5,6–8]^ With regard to environmental factors, monetary rewards^[1,9]^ and social support^[10]^ have been found to impact turnover rate, suggesting that a balanced reward system can lower the turnover rate,^[11,12]^ and strong workplace support contributes to workers’ willingness to continue working.^[11,13]^ These factors have been shown to be interlinked and influenced by issues related to workers jobs and personal lives as well as those related to the organization itself.^[14]^

Furthermore, other factors that are also associated with the turnover rate, such as those regarding children's care in the kindergarten, support for children who have developmental disabilities in the kindergarten, and support from families or caretakers have not been actively studied. Thus, it is also imperative to consider the kindergarten teacher's job specificity, such as unexpectedly caring for children with developmental disabilities, as this may also impact the turnover rate. Thus, this study's primary objective is to investigate the individual and environmental factors that impact kindergarten teachers’ willingness to continue working. Based on their responses, participants were divided into 2 groups: Group 1 consisted of participants willing to work less than 5 years, whereas Group 2 included those who wanted to work for 5 or more years. The findings could contribute toward ameliorating the various drivers of high turnover rates among kindergarten teachers in Japan, where a related shortage is a growing problem.

## 2 Methods

### 2.1 Subjects

**Figure 1:**
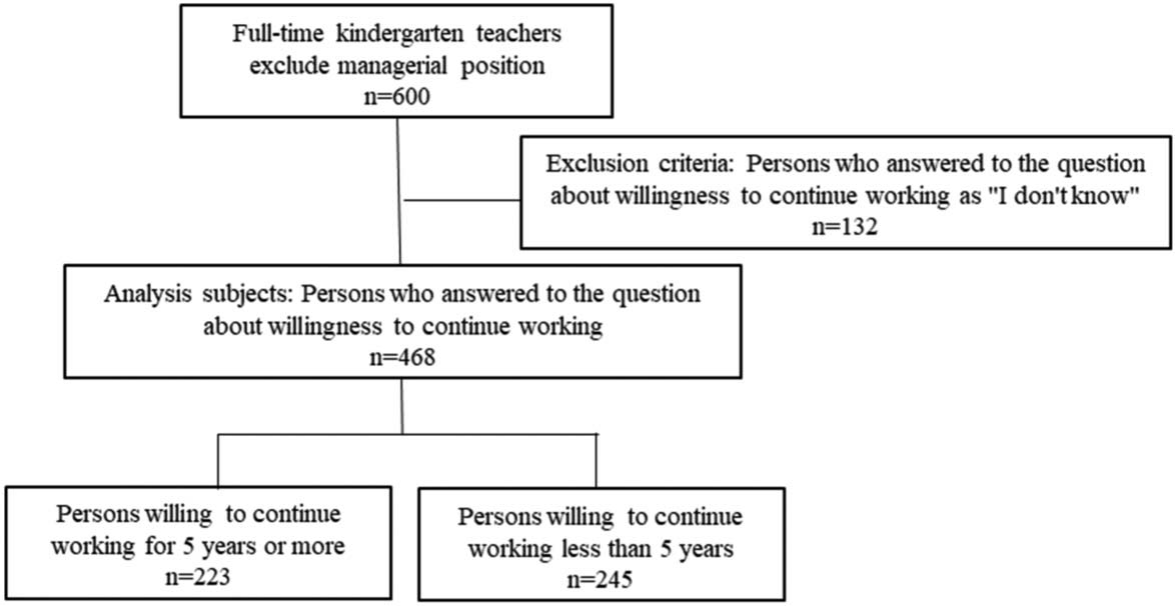
Flow chart of the research. The 468 participants who met the research criteria were divided into 2 groups. The first group included teachers who were willing to continue working for 5 or more years, and the second included those who are willing to work for less than 5 years.

A total of 1137 full-time kindergarten teachers working in kindergartens or authorized childcare institutions in Nagasaki prefecture, excluding those in managerial positions, were recruited to participate in the study. We included only full-time teachers to ensure that part-time workers with fixed-term contracts would not affect the results. From these potential participants, we received 600 completed responses (collection rate: 53%). The study was conducted from April to December 2017. Prior to conducting the analysis, 132 participants whose answer to the question about willingness to continue working was “I do not know” were excluded. Thus, the final sample consisted of 468 participants: 457 female and 10 male teachers; 54%, 22%, 16%, and 8% were in their 20s, 30s, 40s, and over 50, respectively (Fig. 1). Informed consent was obtained from all participants before the study. The study was conducted at the Center for Child Mental Health Care and Education, Nagasaki University. It was approved by the Ethics Committee of Nagasaki University (approval No. 17060864) and complied with the Declaration of Helsinki.^[15]^

### 2.2 Procedure

Participants were recruited from all kindergartens and authorized childcare institutions in Nagasaki, Japan. The study questionnaires took approximately 10 minutes to complete and were answered by participants, who all provided informed consent, in their workplaces. Participants completed the general questionnaire, the Kessler Screening Scale for Psychological Distress (K6), the Utrecht Work Engagement Scale (UWES), and the Social Support Questionnaire (SSQ). All questionnaire responses were self-reported and given anonymously: participants returned their answered questionnaires in sealed envelopes. For the purposes of analysis, participants were divided into 2 groups, those who expressed willingness to continue working in their current position for less than 5 years and those who were willing to work at least 5 years (Fig. 1).

### 2.3 Measures

#### 2.3.1 General questionnaire

The general questionnaire comprised 12 sections: gender, age, family environment, number of children in their families, educational background, work experience, main responsibilities at work, type of workplace, number of colleagues in their workplace, children with disabilities in their class, children who could have developmental disabilities in their class, and willingness to continue working in their present position for 5 years or more. Table 1 reports comparative data from this questionnaire between participants willing to continue working for 5 or more years and those willing to continue working for less than 5 years.

**Table 1:** Comparison between teachers’ willingness to continue working for 5 or more years and less than 5 years.

Variables	Willingness to continue working for 5 or more years n = 223	Willingness to continue working for less than 5 years n = 245	*P*-value
Gender (female/male, n (%))	215/8 (96/4)	242/2 (99/1)	.039^∗^
Age, n (%)			
20 to 29	81 (36)	174 (71)	<.001^∗^
30 to 39	51 (23)	51 (21)	
40 to 49	62 (28)	12 (5)	
50≧	29 (13)	8 (3)	
Family environment, n (%)			
Living alone/Transfer without family	25 (11)	32 (13)	.280
Living with a spouse	97 (43)	27 (11)	.069
Living with children	89 (40)	19 (8)	<.001^∗^
Living with parent	92 (41)	179 (73)	<.001^∗^
Living with spouse's parent	14 (6)	3 (1)	<.001^∗^
Living with brother/sister	33 (15)	77 (31)	<.001^∗^
Living with others	13 (6)	9 (4)	.004^∗^
Number of children in the teachers’ families (mean ± SD)	1.97 ± 0.84	2.00 ± 0.79	.774
Educational background, n (%)			
Bachelor<	163 (73)	149 (61)	.004^∗^
Bachelor≧	59 (26)	96 (39)	
Work experience (years, mean ± SD)	12.37 ± 9.27	5.97 ± 6.11	<.001^∗^
Main responsibilities at work, n (%)			
0-2 years old childcare or other	92 (41)	65 (27)	.001^∗^
3–5 years old childcare or education	131 (59)	180 (73)	
Type of workplace, n (%)			
Public kindergarten	28 (13)	8 (3)	<.001^∗^
Private kindergarten	33 (15)	62 (25)	
Public authorized childcare institution	13 (6)	10 (4)	
Private authorized childcare institution	143 (64)	161 (66)	
Number of colleagues in the workplace (have/none, n (%))	68/143 (30/64)	70/167 (29/68)	.538
Children with disabilities in the class (n, mean ± SD)			
Physical disability	0.10 ± 0.30	0.18 ± 0.38	.242
Hearing disability	0.06 ± 0.23	0.06 ± 0.25	.939
Visual disability	0.07 ± 0.26	0.03 ± 0.18	.278
Intellectual disability	0.19 ± 0.47	0.17 ± 0.46	.896
Developmental disability	0.80 ± 1.13	0.78 ± 0.94	.749
Others	0.22 ± 0.46	0.16 ± 0.45	.459
Children who could have developmental disabilities in the class (mean ± SD)	1.62 ± 2.03	1.59 ± 1.62	.519
Mental health			
K6 < 13, n (%)	213 (96)	202 (82)	<.001^∗^
K6≧13, n (%)	10 (4)	43 (18)	
Work engagement (UWES score, mean ± SD)	63.94 ± 13.50	55.08 ± 13.05	<.001^∗^
Available social support (SSQ number/score, mean ± SD)			
Amount number, mean ± SD	4.08 ± 1.85	3.78 ± 2.17	.109
Satisfaction score, mean ± SD	4.98 ± 0.87	4.90 ± 0.82	.309

Willingness to continue working is significantly affected by the independent variables – gender, age, living with children, living with parent, living with spouse's parent, living with brother/sister, living with others, educational background, main responsibilities at work, type of workplace, work experience, K6 scores, and UWES scores. K6 = The Kessler Screening Scale for Psychological Distress, SSQ = Social Support Questionnaire, UWES = Utrecht Work Engagement Scale.

∗ *P* < .05 by chi-squared test or Mann–Whitney *U* test or *t* test.

#### 2.3.2 Mental health

K6 was used to assess the mental health of the kindergarten teachers. The scale has been used in annual government health surveys in the United States, Canada, and the World Health Organization World Mental Health Surveys.^[16]^ The scale has 6 questions related to the frequency of experiencing psychological distress over a period of 30 days, and the participants were asked to answer on a scale of 5 for each question (0: not at all, 1: a little, 2: sometimes, 3: often, 4: always). High K6 scores indicated a high level of psychological distress. The participants were segregated into 2 categories based on their scores – K6 < 13 or K6 ≥ 13. Scores in the K6 ≥ 13 category indicated that they frequently faced psychological distress.

#### 2.3.3 Work engagement

The UWES was used to assess work engagement, which is the opposite of burnout. Contrary to those who suffer from burnout, engaged employees are energetic, have a sense of effective connection with their work activities, and believe that they can manage the demands of their job well.^[17]^ This segment of the questionnaire had 17 categories, including vigor items (6), dedication items (5), and absorption items (6).^[18]^ The participants were asked to answer on a scale of 6 (1: feel very rarely, 2: feel not too often, 3: feel sometimes, 4: feel often, 5: feel very often, 6: feel always) for each question. High UWES scores indicated high work engagement.

#### 2.3.4 Available social support

The SSQ was used to assess the social support available for kindergarten teachers. The SSQ investigates 2 aspects of social support: the amount of social support a person perceived they have in their life and the degree to which they are personally satisfied.^[19]^ The participants were asked to answer on a scale of 7 (0–5, and more than 6) for aspect, and a scale of 6 (from highly dissatisfied to very satisfied) for aspect. High SSQ numbers and scores indicated that a large amount of social support was available.

### 2.4 Statistical analysis

Chi-square tests were used to examine differences in the categorical variables (gender, age, family environment, educational background, responsibilities at work, type of workplace, number of colleagues at the workplace) between the 2 groups (ie, those willing to continue working for 5 or more years and those willing to continue working for less than 5 years). The Kolmogorov–Smirnov test was used to examine normality for continuous variables. The Mann–Whitney *U* test was used for variables that were not normally distributed (ie, number of children in the participants’ families, disabled children in the class, children who potentially could have developmental disabilities in the class, and K6 scores). Additionally, independent *t* tests were used for normally distributed variables (work experience, UWES scores, and SSQ numbers and scores).

The participants were divided into 2 categories based on the median for UWES scores. Using the data from the analysis of the participants, multiple logistic regression analysis with backward stepwise selection was performed with the willingness to continue working for 5 or more years as the dependent variable. *P*-values, odds ratios (OR), and 95% confidence intervals were calculated. While calculating the multivariate OR, gender, age, family environment (living with a spouse and/or children), main responsibilities at work, type of workplace, K6 scores, and UWES scores were set as covariates. Finally, Spearman rank correlation was used to examine the correlations between factors significantly associated with willingness to continue working. The statistical analysis software SPSS (Version 22.0, IBM, Tokyo, Japan) was used for data analysis. Differences with a *P*-value of <.05 were considered statistically significant.

## 3 Results

The results indicated that the dependent variable (willingness to continue working for 5 or more years) was significantly affected by the independent variables – gender (*P* = .039), age (*P* < .001), living with children (*P* < .001), living with parent (*P* < .001), living with a spouse's parent *(P* *<* .001), living with brother or sister (*P* < .001), living with others (*P* = .004), educational background (*P* = .004), main responsibilities at work (*P* = .001), type of workplace (*P* < .001), work experience (*P* < .001), K6 scores (*P* < .001), and UWES scores (*P* < .001), using the chi-squared test, Mann–Whitney *U* test, or *t* test. There were no significant differences between the other variables (Table 1).

The results of multiple logistic regression analysis with the step-down procedure indicated that the following factors are significantly associated with a higher willingness to continue working for 5 or more years – age, family environment, main responsibilities at work, mental health, and work engagement. The OR of older participants (≥ 40 years of age) was 3.4 times higher than that of younger participants (< 30 years of age). The OR of living with a spouse was 2.4 times higher than that of not living with a spouse. The OR of responsibilities related to care of younger children (up to 2 years old) at work was 2.2 times higher than that of older childcare responsibilities (care and education of children aged 3–5 years). The OR of good mental health (K6 < 13) was 4.5 times higher than high risks of mental health (K6≥13), while that of higher work engagement was 3.4 times higher than lower work engagement (Table 2).

**Table 2:** Multiple logistic regression analysis between willingness to continue working and demographics, K6 scores, and UWES scores.

	Multivariate OR	
Variables	OR (95% CI)	*P*-value
Gender		
Female	1.00 (referent)	
Male	4.81 (0.83–27.90)	.080
Age		
20 to 29	1.00 (referent)	
30 to 39	1.25 (0.69–2.26)	.456
40 to 49	3.41 (1.45–8.01)	.005^∗^
50≧	2.56 (0.97–6.78)	.058
Family environment		
Not living with spouse or children	1.00 (referent)	
Living with a spouse	2.44 (1.21–4.93)	.013^∗^
Living with children	2.15 (0.95–4.91)	.068
Main responsibilities at work		
0 to 2 years old childcare or other	1.00 (referent)	
3 to 5 years old childcare or education	0.47 (0.28–0.79)	.004^∗^
Type of workplace		
Public kindergarten	1.00 (referent)	
Private kindergarten	0.36 (0.12–1.04)	.060
Public authorized childcare institution	0.67 (0.17–2.69)	.570
Private authorized childcare institution	0.39 (0.14–1.09)	.071
Mental health		
K6 < 13	1.00 (referent)	
K6≧13	0.22 (0.09–0.53)	.001^∗^
Work engagement		
UWES < 0.58	1.00 (referent)	
UWES≧0.58	3.44 (2.15–5.50)	<.001^∗^

The following factors are significantly associated with higher willingness to continue working for 5 or more years – age, family environment, main responsibilities at work, mental health, and work engagement. CI = confidence interval, K6 = The Kessler Screening Scale for Psychological Distress, OR = odds ratios, UWES = Utrecht Work Engagement Scale.

∗ *P* < .05 by Multiple logistic regression analysis.

The factors associated with willingness to continue working for less than 5 years are shown in Table 3. The main factor stated was too much overtime work, followed by low salary, and human relations. Additionally, the correlation between the factors associated with working for less than 5 years and factors associated with higher willingness to continue working for 5 or more years are shown in Table 4. The results show that age or family environment were significantly correlated with marriage and health and family problems that indicated a willingness to continue working for less than 5 years. Similarly, older age and living with a spouse were correlated with health and family problems. Moreover, older childcare responsibilities (care and education of children aged 3–5 years) were significantly correlated with too much overtime work, while responsibilities related to care of younger children (up to 2 years old) at work were correlated with differences in childcare/education policy between workplace and oneself, working time/day problems, and health problems. Both good mental health and high work engagement were significantly correlated with marriage, while high risks of mental health and low work engagement were correlated with human relations, differences in childcare/education policy between workplace and oneself, and difficulties taking care of children (Table 4).

**Table 3:** Factors for willingness to continue working for less than 5 years.

Factors for willingness to continue working for less than 5 years n = 245	n (%)
Too much overtime work	124 (51)
Low salary	99 (40)
Human relations	65 (27)
Marriage	62 (25)
Too few holidays	52 (21)
Pregnancy/giving birth	32 (13)
Other reasons	29 (12)
Differences in childcare/education policies between workplace and oneself	27 (11)
Difficulties of procuring childcare/education at work	27 (11)
Difficulties in taking care of children	26 (11)
Difficulties in taking care of guardian	25 (10)
Unsatisfactory welfare benefits	22 (9)
Inadequate levels of satisfaction at work	17 (7)
Working time/day problems	15 (6)
Health problems	11 (4)
Family problems	7 (3)

The factor with the highest percentage was “too much overtime work” followed by “low salary”, and “human relations.”.

**Table 4:** Correlation between factors for willingness to continue working for less than 5 years and factors associated with willingness to continue working for 5 years or more.

(ρ)	Factors for willingness to continue working for 5 years or more				
Factors for willingness to continue working for less than 5 years	Age	Family environment	Main responsibilities at work	K6	UWES
Too much overtime work	−0.051	−0.113	0.261^∗∗^	0.043	0.027
Low salary	−0.073	−0.069	0.104	0.065	−0.111
Human relations	0.082	−0.005	−0.079	0.424^∗∗^	−0.177^∗^
Marriage	−0.174^∗∗^	−0.145^∗∗^	0.073	−0.128^∗^	0.199^∗∗^
Too few holidays	−0.095	−0.023	−0.050	0.019	−0.146^∗^
Pregnancy/giving birth	−0.068	0.062	0.006	−0.153^∗^	0.037
Other reasons	0.107	0.032	−0.009	−0.038	0.151^∗^
Differences in childcare/education policies between workplace and oneself	−0.014	0.043	−0.143^∗^	0.148^∗^	−0.171^∗∗^
Difficulties of procuring childcare/education at work	0.046	0.084	−0.025	0.203^∗∗^	−0.072
Difficulties in taking care of children	−0.071	−0.072	−0.051	0.204^∗∗^	−0.139^∗^
Difficulties in taking care of guardian	0.121	0.054	0.050	0.210^∗∗^	−0.062
Unsatisfactory welfare benefits	0.069	0.117	0.027	0.017	0.057
Inadequate levels of satisfaction at work	0.020	0.006	−0.018	0.140^∗^	−0.102
Working time/day problems	0.019	0.073	−0.001	0.052	−0.071
Health problems	0.216^∗∗^	0.175^∗∗^	−0.138^∗^	0.110	−0.054
Family problems	0.138^∗^	0.174^∗∗^	−0.119	−0.060	0.013

The following factors, namely, marriage, health and family problems, too much overtime work, differences of childcare/education policy between workplace and oneself, working time/day problems, human relations, and difficulties in taking care of children, were correlated with the factors of willingness to continue working for less than 5 years. K6 = The Kessler Screening Scale for Psychological Distress, UWES = Utrecht Work Engagement Scale.

∗ *P* < .05 by Spearman rank correlation.

∗∗ *P* < .01 by Spearman rank correlation.

## 4 Discussion

The present study analyzed the factors associated with the willingness of full-time nonmanagerial kindergarten teachers to continue working. Factors such as being older, living with a spouse, responsibilities related to the care of younger children at work, good mental health, and higher work engagement were significantly associated with a higher willingness to continue working for 5 or more years, while the number of children with developmental disabilities in the class and the number of children who potentially could have developmental disabilities did not show any significant association. A previous survey on the efficacy, self-esteem, social support, and organizational commitment of childcare teachers in Korea suggested that both schools and families are important in teachers’ lives. Events in school can affect their personal lives, and events at home can impact school life.^[20]^ Additionally, a Japanese cross-sectional study suggested that a supportive work environment allows them to continue working even under difficult family circumstances. Regardless of generational characteristics or being influenced by different domestic economic conditions, the psychosocial work environment factors of effort and monetary rewards are generation-common factors^[21]^ that impact intention to work. These results suggest that, in addition to individual factors such as age, teachers’ family and work environment also impact their willingness to continue working, indicating that individual factors and environmental conditions interact.

The primary factors associated with willingness to continue working for less than 5 years were too much overtime, low salary, and human relations. A correlation analysis was performed for factors significantly associated with willingness to continue working. The results suggest that a willingness to continue working for less than 5 years was correlated with marriage among workers who were younger or not living with a spouse, health and family problems among workers who were older or living with a spouse, too much overtime among workers with older childcare responsibilities, and differences in childcare/education policy between workplace and the teacher, and working time/day problems for teachers who were caring for younger children. Furthermore, marriage was correlated with good mental health and high work engagement. In contrast, human relations, differences in childcare/education policy between the workplace and teacher, and difficulties caring for children were correlated with a high risk of mental health issues and low work engagement. However, low salary, one of the strongest drivers of willingness to continue working for less than 5 years, was not correlated with other factors. These findings indicate the necessity of having different strategies based on each factor to lower the turnover rate. Conversely, our results suggest that willingness to continue working is not influenced by the number of disabled children in class but by the perceived difficulties associated with taking care of children.

In terms of individual factors, our results suggest that marriage is correlated with factors influencing the willingness to continue working for less than 5 years among younger teachers, while health and family problems are correlated with willingness to continue working for less than 5 years among older teachers. An Indian cross-sectional survey suggested that a work-life balance that helped employees better manage their work and personal time was a factor that affected job satisfaction.^[16]^ This referred to family-friendly work arrangements and alternative work arrangements, including programs such as flexible work options, paid maternal or paternal leave, and home telecommuting that could increase workers’ satisfaction levels. Additionally, other researchers found that low satisfaction with salaries, decision-making, daily tasks, and work-life balance could decrease workers’ job satisfaction.^[22–24]^ Moreover, a Gambian study suggested that better implementation of stress management plans by the government could have a beneficial impact on the broader psychosocial needs of workers.^[25]^ Individual support systems leading to higher self-esteem and better effort-reward balance for workers may positively influence their well-being.^[26]^ In a randomized controlled trial involving Norwegian workers in private kindergartens, nondirective social support—which focuses on workers’ intrapsychic challenges (eg, the need for restoring self-worth feelings)^[27–29]^–was found to be significantly associated with fewer health problems.^[30]^ Nondirective social support often leads to improvements in positive health behavior, health outcomes, life satisfaction, self-esteem, and hope and optimism.^[28–30]^ Thus, workplace welfare benefits and individual support services could be key elements that could improve job satisfaction and mental health and motivate teachers to continue working after marriage or as problems related to health and family care emerge.^[31–33]^

In terms of environmental factors, our study suggests that too much overtime work is correlated to workers with older childcare responsibilities (care and education of children aged 3–5 years), indicating that teachers working with older children have a greater workload than those caring for younger children (up to 2 years old). Furthermore, human relations, differences in childcare/education policies between workplace and teachers, and difficulties with caring for children were correlated with a high risk of mental health issues and low work engagement. At the same time, low salary, which was one of the highest factors cited for willingness to continue working for less than 5 years, was not correlated with any factor. Prior research suggested that, because of the detrimental impact of heavy workload-related pressures,^[14]^ supervisors should attempt to help workers maintain the right balance between work demands, work pace, and workload.^[34]^ A survey conducted on the psychosocial risks and occupational functioning among workers in Poland indicated that greater involvement with their work might reduce the stress and role conflict and could also increase job satisfaction. Low job satisfaction is considered an emotional response to job strain.^[35]^ Being part of a workplace provides workers with good access to social support, which may play an important role in promoting health.^[30]^ As previous studies have suggested, efforts should be made to improve conditions in both work-related and outside-work areas. Policymakers should continue to improve reward systems, the construction of infrastructure, and promotion systems, and should pay more attention to kindergarten teachers’ lives outside work to ensure their living needs are met.^[4]^ Accordingly, work supervisors should control conditions such as workload and overtime and seek workers’ strong agreement with the childcare/education policies in the workplace. These initiatives may lead to lower turnover. In addition, providing counseling support for human relations factors, including taking care of children and good interpersonal relationships, can lead to better mental health,^[33,35–37]^ job satisfaction,^[38]^ and client satisfaction.^[14]^ Some studies have reported that difficult workplace relationships can lead to workplace stress across occupations.^[39]^ Hence, the development of programs – such as offering workplace counselling – may reduce problems related to human relations, with potentially positive effects on the teachers’ mental health and job satisfaction levels. Additionally, training programs and educational opportunities may help teachers determine how to best take care of children.

### 4.1 Research limitations

In this study, all the participants were recruited from a single prefecture in Japan, and it is unclear if the results can be generalized and are applicable to kindergarten teachers in different prefectures or other countries. Future research should include participants working in different prefectures or other countries. Despite these limitations, the findings regarding factors responsible for willingness to continue working among kindergarten teachers provide meaningful new insights for addressing work environment issues and the shortage of kindergarten teachers in Japanese childcare institutions.

## 5 Conclusions

The findings of this cross-sectional study suggest that individual factors and environmental conditions can influence the willingness to continue working among kindergarten teachers. The results suggest that older age, living with a spouse, responsibilities related to care of younger children at work, better mental health, and higher work engagement are significantly associated with a higher willingness to continue working. Furthermore, marriage, health, and family problems, too much overtime work, differences in childcare/education policy between workplace and teacher, working time/day problems, human relations, and difficulties taking care of children are significantly correlated with these factors. Accordingly, welfare benefits and individual support systems in the workplace or provided by work supervisors could be key elements to encourage teachers to continue working and improve their job satisfaction, mental health, and well-being. Balanced work conditions and workers’ high agreement with workplace childcare/education policy may lead to a reduction in turnover. Programs such as relationship counseling could have a positive impact on teachers’ mental health and job satisfaction. This study's findings may contribute toward addressing the high turnover rate among kindergarten teachers in Japan.

## Acknowledgments

This study did not receive any specific grant from funding agencies in the public, commercial, or not-for-profit sectors. The authors would like to express their appreciation to the participants of this study.

## Author contributions

MM contributed to literature search, data analysis, statistical analysis, manuscript preparation, manuscript editing, and manuscript review. GT, SS, YY, AI, and II contributed to the research concept, design, and the definition of intellectual content. AT contributed to the research concept, design, and data acquisition. TH and SH contributed to statistical analysis. RI contributed to the research concept, design, definition of intellectual content, literature search, statistical analysis, manuscript editing, and manuscript review.

**Conceptualization:** Moemi Matsuo, Goro Tanaka, Akiko Tokunaga, Susumu Shirabe, Yuri Yoshida, Akira Imamura, Izumi Ishikawa, Ryoichiro Iwanaga.

**Data curation:** Moemi Matsuo, Sumihisa Honda.

**Formal analysis:** Moemi Matsuo, Toshio Higashi, Sumihisa Honda, Ryoichiro Iwanaga.

**Investigation:** Moemi Matsuo, Akiko Tokunaga, Ryoichiro Iwanaga.

**Methodology:** Moemi Matsuo, Goro Tanaka, Akiko Tokunaga, Susumu Shirabe, Yuri Yoshida, Akira Imamura, Izumi Ishikawa, Ryoichiro Iwanaga.

**Project administration:** Moemi Matsuo.

**Software:** Toshio Higashi.

**Supervision:** Ryoichiro Iwanaga.

**Writing – original draft:** Moemi Matsuo.

**Writing – review & editing:** Moemi Matsuo, Sumihisa Honda, Ryoichiro Iwanaga.
